# P-401. Cost-Effectiveness of Different Screening Strategies to Control the Spread of Carbapenemase-producing Organisms

**DOI:** 10.1093/ofid/ofae631.602

**Published:** 2025-01-29

**Authors:** Hwee Pin Phua, Darius Beh, Brenda Ang, Wei-Yen Lim

**Affiliations:** Tan Tock Seng Hospital, Singapore, Not Applicable, Singapore; Tan Tock Seng Hospital, Singapore, Not Applicable, Singapore; Tan Tock Seng Hospital, Singapore, Not Applicable, Singapore; Tan Tock Seng Hospital, Singapore, Not Applicable, Singapore

## Abstract

**Background:**

The transmission of carbapenemase-producing organisms (CPO) in healthcare facilities is associated with increased morbidity and mortality. Recommended control measures include early detection of asymptomatic carriers coupled with isolation and contact precautions. Such measures can be costly and need to be balanced against the disruption of hospital operations. We assessed the effectiveness of different screening strategies at averting potential CPO infections and the corresponding cost savings.

**Figure:**
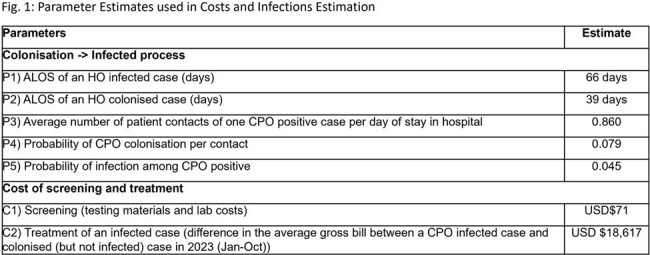

**Methods:**

A basic infectious disease transmission model was used to characterize colonization and transmission with inputs parameterized based on 2023 data from a 1700-bed academic teaching hospital (Fig. 1). Three strategies were compared to the baseline viz. high-risk screening (HRS) but at 70% compliance (based on previous audits) (Fig. 2): (i) HRS at 100% compliance (cHRS); Universal screening on admission (US); Long-stayer screening (LSS) of all inflight patients at every 10 days of stay till 30 days (where applicable) on top of HRS. Once CPO carriage is identified, we assumed no onward transmission, as per institution policy, timely isolation with contact precautions would be implemented. Outcomes included the monthly number of CPO infections and estimated cost of screening (using rapid PCR) and treatment. All costs were converted to US dollars at an exchange rate of USD$1 = SGD$1.343.

**Figure:**
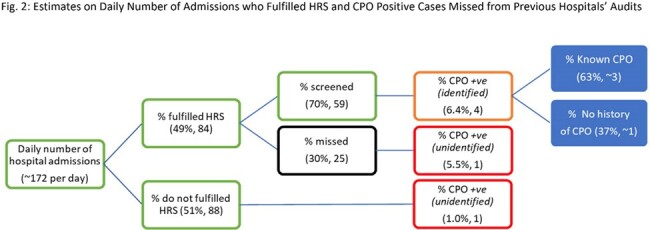

**Results:**

The baseline strategy resulted in 17 infections/month with an estimated cost of USD$443,000 (Fig. 3). Among the three strategies, cHRS gave the best value with potential cost savings USD$39,000/month and 5 infections averted/month. In comparison, LSS in addition to HRS would similarly avert 5 infections/month but at an estimated cost of USD$74,000 (Fig. 4). For US, 8 infections/month would be averted at a cost of USD$79,000 (Fig. 4).

**Figure:**
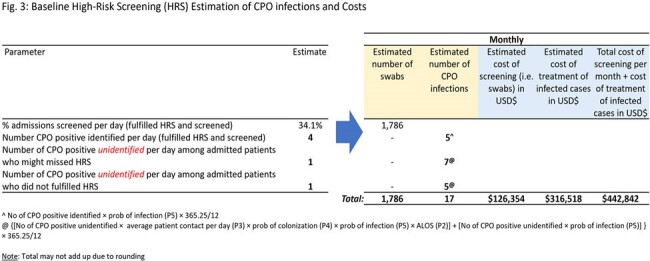

**Conclusion:**

Improving compliance to HRS was the most cost-effective option to limit the spread of CPO.

**Figure:**
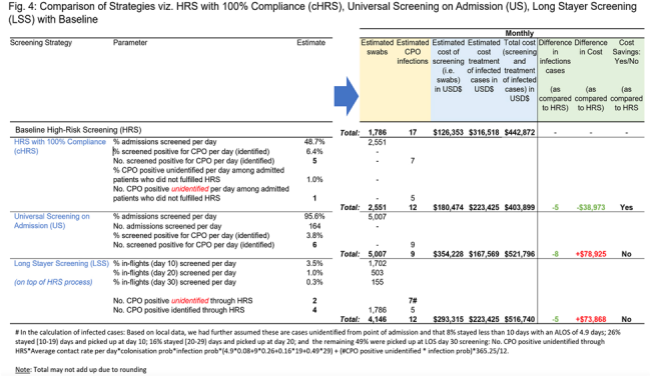

**Disclosures:**

**Darius Beh, MBBS (S'pore) MMed (Int Med) MRCP (UK)**, AstraZeneca: Conference Sponsorship|Gilead: Conference Sponsorship

